# Return to work in sick-listed cancer survivors with job loss: design of a randomised controlled trial

**DOI:** 10.1186/s12885-015-1051-1

**Published:** 2015-02-18

**Authors:** Martine P van Egmond, Saskia FA Duijts, Sylvia J Vermeulen, Allard J van der Beek, Johannes R Anema

**Affiliations:** 1 Department of Public and Occupational Health, VU University Medical Center, EMGO+ Institute for Health and Care Research, Van der Boechorststraat 7, 1081 BT Amsterdam, the Netherlands; 2 Dutch Research Center for Insurance Medicine, AMC-UMCG-UWV-VUmc, Amsterdam, the Netherlands; 3 Department of Psychosocial Research and Epidemiology, The Netherlands Cancer Institute, Amsterdam, the Netherlands

**Keywords:** Cancer, Oncology, Job, Return to work, Intervention, Randomised controlled trial, Study protocol, Unemployment

## Abstract

**Background:**

Despite long-term or permanent health problems, cancer survivors are often motivated to return to work. For cancer survivors who have lost their job, return to work can be more challenging compared to employed survivors, as they generally find themselves in a more vulnerable social and financial position. Cancer survivors with job loss may therefore be in need of tailored return to work support. However, there is a lack of return to work intervention programs specifically targeting these cancer survivors. The number of cancer survivors with job loss in developed countries is rising due to, amongst others, increases in the incidence and survivor rate of cancer, the retirement age and the proportion of flexible employment contracts. Hence, we consider it important to develop a tailored return to work intervention program for cancer survivors with job loss, and to evaluate its effectiveness compared to usual care.

**Methods/Design:**

This study employs a two-armed randomised controlled trial with a follow-up period of 12 months. The study population (n = 164) will be recruited from a national sample of cancer survivors (18–60 years), who have been sick-listed for 12–36 months. Participants will be randomised by using computerized blocked randomisation (blocks of four). All participants will receive usual care as provided by the Dutch Social Security Agency. Additionally, participants in the intervention group will receive a tailored return to work intervention program, which includes vocational rehabilitation and supportive psychosocial components, as well as (therapeutic) placement at work. The primary outcome measure is duration until sustainable return to work; the secondary outcome measure is rate of return to work. Other parameters include, amongst others, fatigue, coping strategy and quality of life. We will perform Cox regression analyses to estimate hazard ratios for time to sustainable return to work.

**Discussion:**

The hypothesis of this study is that a tailored approach for cancer survivors with job loss is more effective, regarding return to work, compared to usual care. The results of this study will provide insight into the ways in which return to work can be facilitated for cancer survivors with job loss.

**Trial registration:**

Netherlands Trial Register: NTR3562.

## Background

Cancer is increasingly perceived as a chronic disease with long-term or permanent health problems [1-3]. Multiple studies report cancer survivors (CSs) to experience, for example, fatigue, depression or functional impairments long after treatment has been completed [4-6]. As a result, CSs’ quality of life, daily functioning and labour participation may be affected [7,8]. With regard to labour market participation, studies have shown that CSs are often motivated to return to work (RTW) and that they attribute positive effects to work. That is, CSs have reported that work enables them to regain a sense of normalty and control [9]. Also, RTW reduces avoidable work disability, thereby decreasing the risk of financial loss for both CSs and society [10].

A recent review found that circa 62% of CSs return to work within 12 months of sick leave. Although the majority of CSs eventually returns to work, still a considerable group of CSs does not resume work. Multiple reasons have been reported for CSs not returning to work, most of which can be categorized as cancer-related, psychosocial-related or work-related [11]. Specifically, work-related factors, such as absence of an employment contract, may create barriers for RTW of CSs. For persons with an insecure employment status, in general, the literature clearly shows that they experience a larger (emotional and practical) distance to the labour market, are lower educated and have an increased risk for prolonged sick leave compared to employees [12,13]. Also, in their RTW, CSs with job loss may face unique barriers, such as having to go through job application processes and compete with “healthy” workers for a job. As a result, after cancer, the RTW process for persons with job loss may be different from the RTW process in employed persons.

In the absence of an employer, CSs who have lost their job in the Netherlands may receive sickness or disability benefits from the Dutch Social Security Agency (SSA). If so, they will be supported by a SSA team, which consists of an insurance physician, a reintegration expert and a labour expert. The number of CSs that receive benefits, either temporary or permanent, has increased over the years. For instance, in 2013, 10.2% more CSs were receiving temporary disability benefits and 17.7% more CSs were receiving permanent disability benefits, compared to 2012 [14]. It is expected that the number of CSs with job loss in the occupational age will keep rising, not only in the Netherlands, but also in other Western economy countries, as a result of the increases in the incidence and survival rate of cancer [15], the retirement age [16], and the proportion of flexible employment contracts [17]. The Dutch SSA has specifically expressed a need for a tailored RTW intervention program for this target group. Current RTW programs are usually aimed at adjusting the current workplace of the CS and negotiating with the CS’s own employer. For CSs with job loss, these programs are not suitable, as they have no workplace or employer (anymore). In addition, it is important to consider that, for CSs with job loss, RTW includes job application processes and starting in a new job that comes with an unfamiliar working environment. Consequently, CSs with job loss are in need of tailored support that targets these specific barriers to RTW.

Therefore, the aim of this study is to develop a tailored RTW intervention program for CSs with job loss, and to study its effectiveness on duration until sustainable RTW in a randomised controlled trial with a follow-up period of 12 months, compared to usual care, as currently provided by the SSA.

## Methods/Design

### Design/setting

This study employs a two-armed non-blinded randomised controlled trial (RCT) with a follow-up period of 12 months. Data will be gathered using questionnaires at baseline, 3, 6 and 12 months post-study entry. Prior to the start of this study, a focus group study with CSs with job loss and/or unemployment experience was conducted, in order to explore barriers and facilitators they experienced with regard to RTW. We used the results from this focus group study to develop the intervention program that is being evaluated in this study. Design and results of the focus group study will be published separately. The CONSORT statement was used to report the design of this study [18]. The study was approved by the Medical Ethical Committee of the VU University Medical Center (VUmc) and the Scientific Committee of the EMGO+ Institute / VUmc.

### Study population

The study population consists of CSs with job loss, aged 18 to 60 years, who are registered at the SSA and who have been sick-listed and receiving sickness or disability benefits in the last 12–36 months. Within the group of CSs with job loss, registered at the SSA, three subtypes of workers can be distinguished: (1) workers whose temporary employment contract ended before or during sick leave; (2) temporary agency workers, and (3) unemployed workers, i.e., these workers had lost their job prior to their cancer diagnosis, and consequently, they received unemployment benefits. After being diagnosed with cancer, their benefits changed from unemployment benefits to sickness or disability benefits.

CSs who have lost their job will be included in this study if they have completed intensive cancer treatment (at least) six weeks prior to the start of this study (based on self-report by the CS), if their health status allows them to participate in the study (based on self-report by the CS) and if they have no comorbidities (e.g., severe psychological or physical conditions, apart from a potential cancer diagnosis) that would interfere with participating in this study (based on report from the CSs’ general practitioner (GP)). In case a CS is invited to participate, but is still receiving, or scheduled to receive, intensive (cancer) treatment (e.g., chemotherapy, radiotherapy, surgery or another type of intensive curative treatment), he/she will be wait-listed for inclusion until (at least) six weeks after completing such treatment(s). Furthermore, CSs will be excluded in case of pregnancy, lack of knowledge of the Dutch language and/or an ongoing conflict with the SSA regarding a sickness or disability benefit claim. Additionally, CSs will be excluded if they are participating, or signed up to be a participant, in a concurrent scientific study and/or re-integration or rehabilitation program aimed at RTW.

### Recruitment of participants

**Figure 1:**
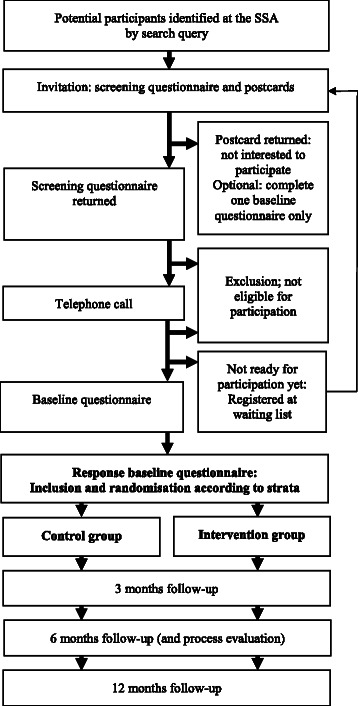
**Participant recruitment flow diagram.**

The process of recruitment is described below and illustrated in the participant recruitment flow diagram (Figure 1).

Potentially eligible participants will be recruited using the national database of the SSA, in which persons applying for sickness or disability benefits are registered. The database contains data regarding demographics, employment status, type of sickness or disability benefit and medical cause, due to which the benefits are granted. A search query will be developed to identify CSs with granted benefits due to a cancer diagnosis. An in-house SSA researcher will use the query to identify potential participants, because of privacy concerns. This SSA researcher will only be involved in this particular part of the recruitment process and in no other parts of the study. Retrospectively, the search query will be used only once to identify CSs who have been sick-listed from 12 to 36 months. Prospectively, with a frequency of twice per month, the search query will be used to identify CSs who have been sick-listed for 12 months. Prospective recruitment will continue for an estimated duration of one and a half years, until the sample size will be reached.

Potential participants will receive an information package from the SSA, which will contain an invitational letter from the chief medical officer from the SSA, a brochure with more detailed information on the study, an informed consent form in which respondents can give their consent to be contacted for information regarding the study, and a short questionnaire. The information package will also include a postcard, by which persons can respond to the researcher whether they are interested in the study or they can provide for declining participation, in case they wish not to participate. Also, on the postcard they can select the option of receiving only the baseline questionnaire, which aims to collect data for comparison between responders and non-responders. Finally, if a person is willing to participate, but only after a few weeks or months, they can select ‘contact me later’ on the postcard. Potential participants who do not return either the short questionnaire, including informed consent form, or the postcard, will receive a reminder letter after two weeks.

The short questionnaire in the information package aims to indicate whether or not the respondent is eligible for participation. Only when a respondent is clearly not eligible for participation in the study, based on this short questionnaire, the researchers will send an informational letter to thank them for their interest in the study. All other respondents will be contacted by telephone to provide additional information about the study procedures and to check if they meet the criteria for participation in the study. After the telephone call, respondents who meet the criteria will receive the baseline questionnaire and a second informed consent form, in which respondents give their consent to participate in the study. Respondents will be offered a choice between receiving the baseline and follow-up questionnaires on paper or via e-mail. If they do not return the baseline questionnaire and/or the informed consent form, then a reminder letter or e-mail will be send after two weeks. When the respondent returns the baseline questionnaire and the informed consent form, he/she will be included in the study and randomly allocated to either the intervention group or the control group. The participant’s GP and the team at the SSA will be notified of the inclusion of the participant in this study and they will receive information about the study. The participant’s file at the SSA will be labelled as ‘research participant’ to facilitate easy recognition by the SSA experts. The GP will be asked to report if the participant has any comorbidities that would interfere with participation (in the intervention program) in this study. In case the participant’s GP feels that a participant’s medical case may be unsuitable for participation in the study, the GP can contact the researchers to deliberate. If necessary, the researchers will organize a joint meeting with the GP and the research team to discuss the CS’s case and achieve consensus about participation.

All participants will be guided by their SSA team according to usual care. Additionally, participants in the intervention group will receive the tailored RTW intervention program. All participants will be asked to complete all questionnaires prior to randomisation (T0), at 3 months (T1), at 6 months (T2) and at 12 months (T3) post-study entry. If a person does not return a follow-up questionnaire, then a reminder letter or e-mail will be sent after two weeks.

### Tailored RTW intervention program

The intervention program was developed as a tailored RTW program in which participants, together with a RTW coach, will decide which needs should be addressed for the participant to RTW. The program was developed by the researchers in cooperation with the Dutch SSA and a national re-integration agency. In the developmental process of the program, we took results from previous studies on RTW for CSs into account [19-21]. For example, this tailored RTW intervention program contains a multidisciplinary approach towards RTW, as the literature showed that multidisciplinary RTW interventions for CSs may be more effective compared to monodisciplinary interventions or usual care [20,21]. Furthermore, we obtained advice from important stakeholders in the field of ‘cancer and work’ in the Netherlands, i.e., medical specialists in oncology, medical social workers, re-integration and vocational rehabilitation agencies, and the Dutch Cancer Patient Movement (Leven met Kanker Beweging) [22]. Finally, we conducted a focus group study with CSs with job loss and with insurance physicians, to identify barriers and facilitators for RTW specifically in CSs with job loss. For example, we discussed which barriers and facilitators CSs with job loss experience in their RTW after cancer. Also, we explored what a suitable duration and intensity level, in terms of frequency of appointments, would be for the intervention program, and in what way the intervention program and the study procedures could be implemented at the SSA. We used the results of the focus group study in the developmental process of the intervention program.

The intervention is consistent with the ‘Dutch Guideline of Oncologic Revalidation (Richtlijn Oncologische Revalidatie)’ [23] and includes elements of a participatory approach, in which the participant will be actively involved in the development, content and execution of his/her RTW plan. Specifically, the participant will be encouraged to actively participate in (1) developing his/her consensus-based tailored RTW plan, (2) coaching on identifying obstacles and creating possibilities for RTW, and (3) exploring possibilities for (therapeutic) return to an actual workplace. The first two steps (developing a RTW plan and coaching) will take place in the participant’s home or at a location nearby, and will be carried out by a re-integration agency, specialized in coaching and support of CSs regarding RTW. The third step (actual placement in a workplace) will be carried out by two job hunting agencies and the participants will travel to the nearest local office(s) of the agencies. The content of the tailored RTW intervention program is, to a certain extent, related to the attitudes-social influences-efficacy model [24]. That is, the first part of the intervention program, i.e., preparation for RTW, relates to behavioral determinants such as attitude and (self-) efficacy. The latter part of the program, e.g., removing barriers for RTW, relates to social influence by involvement of facilitating professionals in the RTW process.

#### Content of the intervention program

**Figure 2:**
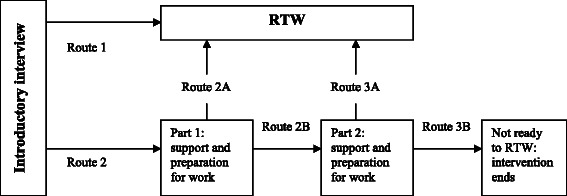
**Routes in the tailored RTW intervention program.**

The tailored RTW intervention program will start with an introductory interview between the participant and an assigned coach from the re-integration agency. Prior to the introductory interview, the participant will be asked to fill out an additional introductory questionnaire. This questionnaire is specifically designed for the coach to obtain insight in the participant’s motivation regarding RTW, needs for additional therapy (e.g., physical and/or psycho-educational), and the skills and knowledge of the participant regarding work and job application processes (e.g., the skill to write letters of application). The results of this questionnaire will be used as input for an introductory interview and to construct a work profile for the participant. During the interview, obstacles and possibilities for RTW and other forms of activities will be identified. Also, the coach and participant will work together during the interview to tailor the participant’s intervention program. There will be several options (or ‘routes’) to tailor the intervention. The possible routes are displayed in Figure 2.

#### Routes in the intervention program

First, the coach will decide, together with the participant, whether or not the participant is ready to RTW, or needs additional support and preparation in order to RTW.

#### Route 1

The participant is ready to RTW at the time of the introductory interview. The coach will contact the researchers to independently assign a job hunting agency to this participant. The job hunter will invite the participant for a meeting to explore job opportunities, thereby taking the participant’s work profile into account. Job hunters will always explicitly ask for a participant’s permission to inform future employers of the participants’ history of cancer. Based on the work profile, the job hunter will start a search for at least two jobs, fitting the profile. These jobs should be for at least three months and may include (1) working in paid employment, or (2) working in temporary employment, i.e., this type of work can be arranged with therapeutic conditions and ongoing benefits. The job hunter will have to find jobs within four weeks after the meeting with the participant. If the job hunter is unable to find these jobs, then the second job hunting agency involved in this study will also be invited to search for jobs for the participant. This involvement includes transfer of confidential information about the participant. The participant will be informed thoroughly about this procedure. Also, the participant can use the established work profile to look for jobs independently, alongside the job search with the job hunter. The total search time for a job will be three months. If both job hunting agencies do not find a suitable job for the participant by then, or if the participant is not able to RTW, then the intervention program will end. The participant will still receive usual care by the SSA during the entire follow-up period.

If the job hunter finds a suitable job, the job hunter will stay in touch with the participant during the remaining period (up to three months) of the intervention program, to monitor whether the participant’s RTW is successful and satisfactory. If applicable, the participant can continue to work in the new job after the intervention program has been completed.

#### Route 2

The participant needs support and preparation to RTW. The coach and participant can explore several topics for coaching. After the introductory interview, the participant will receive four sessions of coaching on chosen themes, e.g., how to deal with fatigue or changed life priorities, how to combine work and family, et cetera. As the intervention program continues, the coach and participant will gradually develop a work profile, which will incorporate the participant’s capabilities, needs and preferences for a workplace. After completing the work profile, the coach and participant will decide whether or not the participant is ready to RTW (Route 2A or Route 2B).

#### Route 2A

The participant is ready to RTW. The participant will continue as described in Route 1. If the participant needs support at the workplace, the participant may receive five additional sessions of coaching while being in the process of RTW.

#### Route 2B

The participant needs more preparation to RTW. The participant will receive five additional sessions of coaching on chosen themes. After this, the coach and participant will again decide whether or not the participant is ready to RTW (Route 3A or Route 3B).

#### Route 3A

If the participant is ready to RTW, the participant will continue as described in Route 1.

#### Route 3B

If the participant is not ready to RTW, the intervention program will be terminated.

In Route 2, both 2A and 2B, the coach can also opt for additional support, for example support from a physical therapist and/or psychologist, in case specific physical and/or mental problems are present. If this is the case, the coach will discuss this with the participant’s expert team at the SSA and/or the participant’s GP.

### Completion of the intervention program

The total duration of the program in the most extensive route (2B followed by 3A) will be six to seven months. Here, the participants will receive preparation to RTW for a maximum of four months, including ten sessions of coaching, job application preparation and possibly recovery support, such as physical therapy, and placement in a workplace for a minimum of three months. In general, we anticipate that duration of the program will vary between participants. Some participants will need the most extensive route, and others may need less support in order to facilitate their RTW. Also, in case of extraordinary circumstances, participants can put their tailored RTW intervention program on hold for a period of one month maximum. The limit of one month was chosen to allow for the program to be tailored to each participant’s needs, while maintaining a relatively similar duration of most participants’ programs. We will monitor the chosen route(s) for each participant. During the program, members of the SSA team, as well as the GP, will be notified of the program’s start, progress and finish. They will receive a copy of the intervention plan and the evaluation report.

### Outcome measures & prognostic factors

Data regarding primary and secondary outcome measures, as well as prognostic factors such as sociodemographic factors (i.e., age, gender, level of education), disease-related and work-related factors will be collected using questionnaires. If possible, additional data regarding usual care will be collected from the participant’s file at the SSA. The primary outcome measure of this study is duration until sustainable RTW after sick leave, calculated as the number of days between the day of randomisation and the first day of sustainable RTW. Sustainable RTW is defined as a period of minimum 28 calendar days, during which the CS is working according to schedule. Work can be either paid work or work resumption with ongoing benefits, e.g., work with therapeutic conditions. Recurrences of sick leave within four weeks of RTW will be considered as belonging to the initial period of sick leave, in accordance with the requirements of the Dutch Sickness Benefits legislation. The secondary outcome measure of this study is rate of RTW, i.e., the proportion of participants in each group that sustainably returns to work. Rate of RTW will be not only obtained from questionnaire data, but also, if possible, from participants’ files at the SSA. We will collect data on a number of prognostic factors:

- **Intention to RTW** will be measured using the ‘Attitudes-Social influence-self-Efficacy’ questionnaire (ASE) [25]. This questionnaire was designed to assess intention to RTW in a study of unemployed persons with common mental disorders. The questionnaire has not been validated. Therefore, its results will be used as an indicator for intention to RTW;
- **Readiness to RTW** will be measured using the Readiness to RTW Scale (RRTW) [26]. The items on the scale are related to the five stages of change described in the Transtheoretical model by Prochaska and DiClemente [27]. We will adjust the Scale to the Dutch situation as there is no validated translation available, and use the results of this scale as an indicator of readiness to RTW, instead of a validated outcome measure;
- **Fatigue** will be assessed with the 13-item self-reported FACT-Fatigue Scale (Version 4) [28]. Fatigue is measured in this questionnaire on a four-point scale (range 0–52). It has a high internal validity with a Cronbach’s alpha of 0.96 and high test-retest reliability (ICC = 0.95) [29];
- **Psychological distress** will be assessed with the Centre for Epidemiological Studies Depression Scale (CES-D) [30-32]. The CES-D is a 20-items questionnaire, measured on a four-point scale. It is designed to measure depressive symptomatology in the general population and has a high reliability with a Cronbach’s alpha of 0.79-0.92 and a test-retest score of 0.90;
- **General participation in society** will be measured using the Utrecht Scale for Evaluation of Revalidation and Participation (User-P) [33]. The User-P scale aims to rate objective and subjective participation in persons with physical disabilities and consists of 31 items in three scales: Frequency, Restrictions, and Satisfaction. Internal consistency of the USER-Participation scales is moderate to good, with Cronbach’s alpha ranging between 0.70 and 0.91. Spearman correlations between these scales range between 0.36 and 0.52. Test-retest reliability of the User-P scales was measured using the intraclass correlation coefficient (ICC). The ICC of the USER-P was 0.65 for the Frequency scale, 0.85 for the Restrictions scale, and 0.84 for the Satisfaction scale;
- **Coping** will be measured using the Utrecht Coping List (UCL) [34]. The UCL has 47 statements covering seven coping strategies, such as active problem solving, seeking social support and depressive reaction, and is scored on a four-point scale. Its reliability varies between 0.43 and 0.89, depending on the subscale used. The test-retest score ranges from 0.45 to 0.85, depending on the subscale used;
- **General health and quality of life** will be assessed with the European Organisation for Research and Treatment of Cancer Quality of Life Questionnaire (EORTC QLQ C-30 version 3.0) [35]. This 30-item list incorporates nine multi-item scales: five functional scales (physical, role, cognitive, emotional and social); three symptom scales (fatigue, pain, nausea and vomiting); and a global health and quality of life scale. The test-retest score has shown to be high for all functional scales with a range of 0.82 to 0.91, and a reliability score over 0.80 for four out of five scales, with cognitive functioning scoring 0.68 [36];
- **Health-related quality of life** will be measured using the EuroQol 5D scale, developed by the EuroQol group (EQ-5D) [37]. The EQ-5D consists of 5 scales: Mobility, Self-care, Usual activity, Pain/Discomfort, and Anxiety/Depression, with a scale of three levels per item (i.e., no problem, some problems, and extreme problems). The EQ-5D is a generic instrument and has been used in cancer research in numerous studies. Furthermore, a visual analogue scale is provided (range 0–100) to assess overall health state;
- **Limitations experienced at work** will be measured in participants who have returned to work during the follow-up period, using the Work Limitations Questionnaire (WLQ) [38]. The WLQ consists of 25 items, which describe four dimensions of limitations: limitations in handling time, physical, mental and interpersonal limitations. The questionnaire was tested in two field trials, and the four scales achieved Cronbach’s alphas of > 0.90.
- **Occupational impact of sleep** will be measured in participants who have returned to work during the follow-up period, using the Dutch Occupational Impact of Sleep Questionnaire (OISQ) [39]. The OISQ consists of 24 items, which aim to assess the effect of sleep quality on work performance. The OISQ has been validated in the Dutch population, correlating highly with other validated sleep questionnaires (coefficients range 0.28 to 0.43, P < 0.0001) and has a high reliability score (Cronbach’s alpha of 0.96). This measure was added as studies have demonstrated that sleep disturbances are common in CSs and that they are related to poorer physical and emotional health, concentration problems (for example at work), and difficulty coping with stress [40].

### Process evaluation

A process evaluation will be conducted to examine the tailored RTW intervention program regarding feasibility, satisfaction, and barriers and facilitators for implementation. The process evaluation will be designed according to the RE-AIM (Reach, Effectiveness, Adoption, Implementation, and Maintenance) framework [41]. The measurements of the process evaluation will be based on: (1) data regarding study procedures and adherence to study protocol, and (2) data collected using additional process evaluation questionnaires, which will be designed separately for participants, team members from the SSA, the re-integration agency and the job hunting agencies.

### Sample size

As a starting point for calculating the sample size, we chose a Hazard Ratio of 2, indicating that the participants in the intervention group RTW twice as quickly compared to the participants in the control group. This Hazard Ratio is based on comparable studies on RTW of workers who are sick-listed and who are receiving sickness benefits [42,43]. Assuming that half of the workers will achieve sustainable RTW during the first 12 months of the follow-up period, and based on a power of (1-β=) 0.80 and a two-sided significance level of 0.05 (α), a sample size of 130 participants (n = 2 × 65) is needed. Based on comparable research, loss to follow-up of 20% is taken into account. This results in 164 participants (n = 2 × 82) to be included in the study. This number of participants seems feasible as samples from the registration database at the SSA indicate that approximately 3000 persons could be invited for participation over a time period of one and a half year.

### Randomisation

Prior to randomisation, we will apply pre-stratification in our inclusion procedure to ensure equal representation of the three subtypes amongst CSs with job loss in our sample; (1) workers whose temporary employment contract ended before or during sick leave; (2) temporary agency workers; and (3) unemployed workers. Randomisation to either the intervention group or control group will be performed on the individual level and will be performed separately for each stratum. Randomisation will be performed by the coordinating researchers (with the exception of the executive researcher), using computerized blocked randomisation by means of the Randomisation Plan Generator [44]. The number of participants in each block will be four, with an allocation ratio of 1:1.

Blinding to the randomisation outcome in this study is not possible due to the nature of the intervention program, in which various stakeholders will need to cooperate to support and guide participants in the intervention group. Furthermore, for practical reasons with regard to usual care, the researchers will have to inform the SSA team and the GP of participants about the inclusion of the participant and the group to which the participant was randomised.

### Co-interventions and compliance

For participants in the control group, we cannot prevent co-interventions, e.g., recovery therapy or support from job hunters, being offered to them, as these interventions can be part of the SSA’s usual care. For participants in the intervention group, we will ask the SSA team not to offer any additional interventions during the period in which the CS participates in our intervention program. During the follow-up period, after the intervention program in this study has been completed, the SSA team may still offer interventions that are available through usual care. We will monitor any co-interventions offered in both groups by asking questions about this in the questionnaires and, if possible, by data from the participants’ files at the SSA.

### Statistical analysis

All statistical analyses will be performed according to the intension-to-treat principle. If necessary, the per-protocol principle will also be applied [45]. Descriptive analyses will be performed to check whether there are relevant differences in the baseline characteristics of the intervention and the control group at baseline. Analyses will be performed on an individual level. All analyses will be performed both crude and adjusted for potential confounders, e.g., gender, age, stratum of participants, or type of cancer. Also, these variables will be checked for effect modification. Scores on the included outcomes measures and parameters in the study will be calculated according to published scoring algorithms. The results of the questionnaires will be compared between both groups at baseline and at 3, 6 and 12 months follow-up. Correction for baseline values will be applied.

The primary outcome measure, duration until sustainable RTW in both groups, will be described using the Kaplan-Meier method. We will use the Cox proportional hazard model to estimate hazard ratios and corresponding 95% confidence intervals for sustainable RTW. Finally, we will also perform multiple regression analysis to determine associations between the primary outcome measure and predictor variables, such as fatigue and coping strategy, in order to identify prognostic factors for RTW in this population. A two-tailed significance level of <0.05 is considered statistically significant in all analyses. All analyses will be performed using SPSS 20.0 [46].

## Discussion

In light of an increasing incidence of cancer and an improving survival rate [47], a rising retirement age [16,48] and a growing number of temporary employment contracts within Western labour markets [17], it is expected that the number of CSs have who lost their job in the working age will increase. CSs with job loss may experience unique challenges in their RTW process, compared to employed CSs, e.g., competition with “healthy” individuals for a job, lack of a workplace to return to and lack of social support from colleagues or an employer. Therefore, they may be in need for a tailored approach for RTW. This study aims to evaluate the effectiveness of a tailored RTW intervention program on duration until sustainable RTW of CSs with job loss, compared to usual care.

### Methodological considerations regarding the study design

There are several methodological aspects of this study that can be considered. One of the main strengths of this study is that, in the developmental process of the tailored RTW intervention program, we have incorporated (1) ideas and perspectives of a large variety of stakeholders in the fields of cancer, work and insurance medicine in the Netherlands, (2) results from previous international studies on RTW for CSs, and (3) results from a qualitative focus group study on barriers and facilitators for RTW in CSs with job loss. Another strength of this study is that we will evaluate the effectiveness of the tailored RTW intervention program with a RCT design, and that we will conduct a process evaluation alongside the RCT. Furthermore, the procedures for this study were developed in accordance with the Dutch SSA. This will facilitate implementation of the study protocol, particularly the recruitment protocol, at the SSA.

There are also several limitations to be considered with regard to the study design. First of all, this is the first study to be conducted that incorporates a RTW intervention for CSs with job loss. Therefore, in developing the tailored RTW intervention program, we had to rely on indirect evidence from studies on RTW for employed CSs, and adjust this information, taking into account our knowledge of the situation of CSs with job loss, which is a subjective process. We did not take other intervention studies on RTW in persons with chronic diseases (other than cancer) into account in developing the tailored RTW intervention program. Still, the tailored RTW intervention program is based on elements of the attitudes-social influences-efficacy model [24], which indicates that there is, to some extent, a scientific basis for the content of the program.

Ideally, this study design would have incorporated a pilot phase, in which we could evaluate whether the intervention program would be acceptable and sufficiently tailored to the needs of CSs with job loss. In addition, a pilot study would have enabled us to discover potential implementation problems for the intervention program beforehand and to evaluate whether or not the chosen recruitment strategy for the RCT would be feasible and successful. Unfortunately, we were not able to carry out a pilot phase in this study.

With regard to the sample size, we chose a Hazard Ratio of 2, which is not uncommon for studies in (moderately) comparable populations. Still, this number might be optimistic in terms of the anticipated results. Another limitation is that this study does not use blinding, i.e., we have to disclose to the participant’s RTW team at the SSA whether the participant is in the intervention group or the control group. This could potentially lead to awareness in the SSA teams about their care being evaluated, which could result in a different type of usual care provided to the intervention group compared to the control group. Finally, blinding could prevent contamination of the control group, but as this study recruits 82 participants for the intervention group on a national level, the researchers estimate the risk of contamination of the control group marginal. Potentially, increased awareness of RTW could lead to participants in the control group employing RTW activities on their own, which they might not have done if they were not participating in the study. Enhanced RTW activities in the control group could distort a potential effect of the tailored RTW intervention program and lead to an underestimation of any effect that will be measured between the intervention group and the control group. Finally, it is not unlikely that CSs with job loss feel disappointed in the labour market (due to job loss) or the social security system (due to a lack of appropriate RTW interventions), which may influence their willingness to participate in an experimental study that offers a RTW intervention program. This could potentially lead to selection bias in our sample of participants.

### Implications of study findings for research

The results of this study will contribute to the literature by providing insight into the RTW process of CSs with job loss and the ways in which RTW can be facilitated for them. In a broader perspective, the results of this study may change the way the RTW process of CSs is generally studied. To this day, the literature on RTW does not distinguish CSs based on work-related factors, such as working status or type of employment contract, but rather distinguishes CSs on medical factors, such as type of diagnosis. If the results of this study demonstrate a positive effect in favor of a tailored approach for CSs with job loss, then opportunities may be created to develop future RTW interventions tailored to work-related factors, e.g., employment status, present in CSs. Possibly, this approach may also be applied to persons with job loss with other (chronic) conditions, in order to facilitate their RTW.

### Implications of study findings for practice

This study may demonstrate that it is effective to tailor RTW support for CSs to work-related factors, such as employment status, at least in Western economy countries. If so, policymakers should find ways to implement tailored RTW intervention programs for CSs with job loss. This study may also have a positive impact on the increasing burden of sickness and disability benefits. As more CSs may return to work as a result of a tailored RTW intervention program, the number of CSs receiving benefits will decrease. As benefits are indirectly provided by the tax payers in Western economy countries, society as a whole could potentially profit from a higher number of CSs returning to work.

### Conclusion

There is a gap in the literature regarding the RTW process of CSs who have lost their job. We hypothesize that CSs with job loss benefit from a tailored approach regarding RTW support, as a result of unique challenges, e.g., lack of a current job, going through job application processes, competing with “healthy” individuals for a job and having a large emotional and practical distance to the labour market. This study aims to facilitate sustainable RTW for CSs with job loss, by offering a tailored RTW intervention program and evaluate its effectiveness compared to usual care. Results of this study will be available in 2016.

## Abbreviations

CS(s): Cancer survivor(s)
GP: General Practitioner
RCT: Randomized controlled trial
RTW: Return to work
SSA: Social security agency
VUmc: VU University Medical Center

## References

[1] Gosain R, Miller K (2013). Symptoms and symptom management in long-term cancer survivors. Cancer J.

[2] Yi J, Kim MA, Tian T (2014). Perceived long-term and physical health problems after cancer: adolescent and young adult survivors of childhood cancer in Korea. Eur J Oncol Nurs.

[3] Signaleringscommissie Kanker, KWF kankerbestrijding [Dutch Cancer Society]. Cancer in the Netherlands: trends, forecasts and implications for health care / Kanker in Nederland. Trends, prognoses en implicaties voor zorgvraag. 2004.

[4] Carlson LE, Angen M, Cullum J, Goodey E, Koopmans J, Lamont L (2004). High levels of untreated distress and fatigue in cancer patients. Br J Cancer.

[5] Hewitt M, Rowland JH, Yancik R (2003). Cancer survivors in the United States: age, health, and disability. J Gerontol A Biol Sci Med Sci.

[6] Karki A, Simonen R, Malkia E, Selfe J (2005). Impairments, activity limitations and participation restrictions 6 and 12 months after breast cancer operation. J Rehabil Med.

[7] Harrington CB, Hansen JA, Moskowitz M, Todd BL, Feuerstein M (2010). It’s not over when it’s over: long-term symptoms in cancer survivors–a systematic review. Int J Psychiatry Med.

[8] Munir F, Yarker J, McDermott H (2009). Employment and the common cancers: correlates of work ability during or following cancer treatment. Occup Med (Lond).

[9] Peteet JR (2000). Cancer and the meaning of work. Gen Hosp Psychiatry.

[10] Lauzier S, Maunsell E, Drolet M, Coyle D, Hebert-Croteau N, Brisson J (2008). Wage losses in the year after breast cancer: extent and determinants among Canadian women. J Natl Cancer Inst.

[11] Mehnert A (2011). Employment and work-related issues in cancer survivors. Crit Rev Oncol Hematol.

[12] Benach J, Amable M, Muntaner C, Benavides FG (2002). The consequences of flexible work for health: are we looking at the right place?. J Epidemiol Community Health.

[13] Vermeulen SJ, Tamminga SJ, Schellart AJ, Ybema JF, Anema JR (2009). Return-to-work of sick-listed workers without an employment contract–what works?. BMC Public Health.

[14] Uitvoeringsinstituut Werknemersverzekeringen [Dutch Institute for Employee Benefit Schemes] (2013). Quantitative Reports of Statistics 2013/UWV Kwantitatieve Informatie.

[15] Bray F, Jemal A, Grey N, Ferlay J, Forman D (2012). Global cancer transitions according to the Human Development Index (2008–2030): a population-based study. Lancet Oncol.

[16] Hoppers F. Increase In State Pension Age From 1 January 2013. http://legalknowledgeportal.com/2012/10/16/increase-in-state-pension-age-from-1-january-2013/ . 16-10-2012. Dirkzwager advocaten & notarissen N.V., Arnhem, The Netherlands.

[17] Benach J, Gimeno D, Benavides FG, Martinez JM, Torne MM (2004). Types of employment and health in the European union: changes from 1995 to 2000. Eur J Public Health.

[18] Schulz KF, Altman DG, Moher D (2011). CONSORT 2010 statement: updated guidelines for reporting parallel group randomised trials. Int J Surg.

[19] Tamminga SJ, de Boer AGEM, Verbeek JHAM, Frings-Dresen MHW (2010). Return-to-work interventions integrated into cancer care: a systematic review. Occup Environ Med.

[20] de Boer AG, Taskila T, Tamminga SJ, Frings-Dresen MH, Feuerstein M, Verbeek JH (2011). Interventions to enhance return-to-work for cancer patients. Cochrane Database Syst Rev.

[21] de Boer AGEM, Frings-Dresen MHW (2009). Employment and the common cancers: return to work of cancer survivors. Occup Med (Lond).

[22] Dutch Cancer Patient Movement (Leven met Kanker Beweging). https://www.kanker.nl/organisaties/levenmetkanker-beweging/2186-homepage. 2014.

[23] Richtlijn Oncologische Revalidatie [http://www.oncoline.nl/index.php?pagina=/richtlijn/item/pagina.php&id=32518&richtlijn_id=765&tab=2]. 2011.

[24] Vries HD, Backbier E, Kok G, Dijkstra M (1995). The Impact of social influences in the context of attitude, self-efficacy, intention, and previous behavior as predictors of smoking onset1. J Appl Soc Psychol.

[25] van Oostrom SH, Anema JR, Terluin B, de Vet HCW, Knol DL, van Mechelen W (2008). Cost-effectiveness of a workplace intervention for sick-listed employees with common mental disorders: design of a randomized controlled trial. BMC Public Health.

[26] Franche RL, Corbiere M, Lee H, Breslin FC, Hepburn CG (2007). The Readiness for Return-To-Work (RRTW) scale: development and validation of a self-report staging scale in lost-time claimants with musculoskeletal disorders. J Occup Rehabil.

[27] Prochaska JO, DiClemente CC (1983). Stages and processes of self-change of smoking: toward an integrative model of change. J Consult Clin Psychol.

[28] Webster K, Cella D, Yost K (2003). The Functional Assessment of Chronic Illness Therapy (FACIT) Measurement System: properties, applications, and interpretation. Health Qual Life Outcomes.

[29] Chandran V, Bhella S, Schentag C, Gladman DD (2007). Functional assessment of chronic illness therapy-fatigue scale is valid in patients with psoriatic arthritis. Ann Rheum Dis.

[30] Radloff LS (1977). The CES-D Scale. Applied Psychological Measurement.

[31] Schroevers MJ, Sanderman R, van Sonderen E, Ranchor AV (2000). The evaluation of the Center for Epidemiologic Studies Depression (CES-D) scale: Depressed and Positive Affect in cancer patients and healthy reference subjects. Qual Life Res.

[32] Bouma J, Ranchor A, Sanderman R, van Sonderen E (2012). Het meten van symptomen van depressie met de CES-D: een handleiding.

[33] van der Zee CH, Priesterbach AR, van der Dussen L, Kap A, Schepers VPM, Visser-Meily JMA (2010). Reproducibility of three self-report participation measures: The ICF Measure of Participation and Activities Screener, the Participation Scale, and the Utrecht Scale for Evaluation of Rehabilitation-Participation. J Rehabil Med.

[34] Scheurs PJG, van de Willige G, Brosschot JF, Tellegen B, Graus GMH (1993). De Utrechtse Copinglijst: UCL.

[35] Hjermstad MJ, Fossa SD, Bjordal K, Kaasa S (1995). Test/retest study of the European Organization for Research and Treatment of Cancer Core Quality-of-Life Questionnaire. J Clin Oncol.

[36] Schwarz R, Hinz A (2001). Reference data for the quality of life questionnaire EORTC QLQ-C30 in the general German population. Eur J Cancer.

[37] Group EQ (1990). EuroQol-a new facility for the measurement of health-related quality of life. HealthPolicy.

[38] Lerner D, Amick BC, Rogers WH, Malspeis S, Bungay K, Cynn D (2001). The Work Limitations Questionnaire. Med Care.

[39] Verster JC, David B, Morgan K, Olivier B (2008). Validation of the Dutch Occupational Impact of Sleep Questionnaire (OISQ). Ind Health.

[40] Davidson JR, MacLean AW, Brundage MD, Schulze K (2002). Sleep disturbance in cancer patients. Soc Sci Med.

[41] Glasgow RE, Vogt TM, Boles SM (1999). Evaluating the public health impact of health promotion interventions: the RE-AIM framework. Am J Public Health.

[42] Vermeulen SJ, Anema JR, Schellart AJM, van Mechelen W, van der Beek AJ (2010). Cost-effectiveness of a participatory return-to-work intervention for temporary agency workers and unemployed workers sick-listed due to musculoskeletal disorders: design of a randomised controlled trial. BMC Musculoskelet Disord.

[43] Lambeek LC, van Mechelen W, Knol DL, Loisel P, Anema JR (2010). Randomised controlled trial of integrated care to reduce disability from chronic low back pain in working and private life. BMJ.

[44] Dallal, GE. Randomization Plan Generator. 16-7-2008.

[45] Heritier SR, Gebski VJ, Keech AC (2003). Inclusion of patients in clinical trial analysis: the intention-to-treat principle. Med J Aust.

[46] SPSS Inc. 2009. Chicago, Illinois, USA.

[47] Ferlay J, Soerjomataram I, Dikshit R, Eser S, Mathers C, Rebelo M (2014). Cancer incidence and mortality worldwide: Sources, methods and major patterns in GLOBOCAN 2012. Int J Cancer.

[48] Reichert SJM (2014). The Dutch Pension System, an overview of the key aspects.

